# HIV awareness, pre-exposure prophylaxis perceptions and experiences among people who exchange sex: qualitative and community based participatory study

**DOI:** 10.1186/s12889-022-14235-0

**Published:** 2022-10-01

**Authors:** Yasaswi Kislovskiy, Sarah Erpenbeck, Jamie Martina, Courtney Judkins, Elizabeth Miller, Judy C. Chang

**Affiliations:** 1 Department of OB/GYN and Women’s Institute, Drexel University College of Medicine, Allegheny Health Network, 4800 Friendship Ave, Pittsburgh Pennsylvania, PA USA; 2 Magee-Womens Research Institute (MWRI), Pittsburgh, PA USA; 3 University of Pittsburgh School of Medicine, Pittsburgh, PA USA; 4 Graduate School of Public Health, University of Pittsburgh, Pittsburgh, PA USA; 5 Division of Adolescent and Young Adult Medicine, UPMC Children’s Hospital of Pittsburgh, Pittsburgh, PA USA; 6 Department of Obstetrics, Gynecology, and Reproductive Sciences, University of Pittsburgh School of Medicine, Pittsburgh, PA USA

**Keywords:** Exchange sex, Sex work, HIV risk, Pre-exposure prophylaxis, Qualitative research, Community based participatory research

## Abstract

**Background:**

People who exchange sex for money, favors, goods or services, combat higher risk of acquiring sexually transmitted diseases (STDs) and human immunodeficiency virus (HIV). Understanding barriers to STD and HIV related healthcare from the perspective of this stigmatized and marginalized community may improve access to sexual health services including pre-exposure prophylaxis (PrEP).

**Methods:**

We used community-partnered participatory and qualitative methods to conduct anonymous one-on-one interviews with people who exchange sex to understand their perspectives and experiences related to pre-exposure prophylaxis (PrEP) to prevent HIV acquisition. We conducted twenty-two interviews and coded them to perform thematic analysis.

**Results:**

We identified five themes: (1) Appreciation of HIV risk and prevention strategies grew from information accumulated over time. (2) PrEP information came from a variety of sources with mixed messages and uncertain credibility. (3) Decision-making about use of PrEP was relative to other behavioral decisions regarding exchange sex. (4) The multi-step process of obtaining PrEP presented multiple potential barriers. (5) Healthcare providers were seen as powerful facilitators to PrEP utilization.

**Conclusions:**

Our findings suggest that PrEP education and care needs to be made more relevant and accessible to individuals who exchange sex.

**Supplementary Information:**

The online version contains supplementary material available at 10.1186/s12889-022-14235-0.

## Background

People who have sex in exchange for money, favors, goods or services, face disproportionately high risk for acquisition of human immunodeficiency virus (HIV) and sexually transmitted diseases (STDs) due to socio-structural environments and laws that confer ongoing risks [1–6]. These individuals also face barriers to existing strategies for prevention, such as condom use, testing and treatment, or pre-exposure prophylaxis (PrEP) [1, 7–10]. Stigmatization, marginalization, and criminalization further disincentivizes people who exchange sex from expressing their opinions on key topics and policies that can affect them [5, 6, 11]. In order to overcome barriers and provide person-centered HIV prevention services for people who exchange sex, we need to understand the needs, desires, and experiences of people who exchange sex.

Famously, initial pre-exposure prophylaxis efficacy trials among sex workers in Cambodia, Nigeria, Thailand and Malawi were halted or were refused IRB approval for ethical issues such as lack of community engagement and safe-keeping [12, 13]. Later PrEP trials showed efficacy in HIV prevention but did not include sex workers [14, 15]. A demonstration project among sex workers in India utilized a community-led approach and showed 97.7% (627 of 647 participants) utilization of PrEP over 15 months and no HIV seroconversions [16]. Facilitators of PrEP utilization in this study included community leaders with consistent and accurate prevention counseling, demonstration of PrEP ingestion by community leaders, and access to full resources such as condoms and a community bank for all members of the sex worker group whether or not they took PrEP [17]. In the United States, among female participants of a safe syringe program in Philadelphia who initiated PrEP, 71.6% (68 of 95 participants) engaged in transactional sex [18]. At 24 weeks after initiating PrEP, only 44.2% (42 of 95 participants) continued utilizing PrEP [18]. Participants discussed complex needs that compete with PrEP utilization such as housing or food [18, 19]. Further work is needed to understand PrEP interest, access, preferences and experiences in the United States population of people who exchange sex, and this work must integrate community voices as integral to future HIV prevention efforts.

Our study utilized a community-partnered participatory approach with qualitative methods to understand the experiences and perspectives of individuals who exchange sex related to PrEP. We used a community partnered approach to reduce the risk of re-traumatization while interviewing participants, and to combat power dynamics that may obscure true feelings and beliefs [20]. Using anonymous semi-structured interviews and assistance from community partners, we describe the experiences and perspectives related to PrEP from the perspective of people who exchange sex.

## Methods

We employed community-partnered participatory methods to design and conduct this study. A community-partnered approach collaborates with community members and organizations in order to improve study design, recruitment, and data analysis [21]. Partnering with community can minimize the risk of harm from medical research and the risk of re-traumatization when discussing vulnerable and sensitive topics [22]. Community partners were from Pittsburgh and included individual members of Sex Workers Outreach Project (SWOP) Pittsburgh chapter, New Voices for Reproductive Justice (a Pittsburgh and Philadelphia based Reproductive Justice organization), PERSAD Center and Allies for Sexual Health and Well-being (two LGBTQIA oriented health clinics), Pittsburgh Action Against Rape (support center including legal and mental health services for survivors of intimate partner violence), and Positive Pathways (rehabilitation services for women who have received prostitution charges). We invited community partners to review the study objectives, study design and qualitative interview materials including the semi-structured interview guide. Partners also assisted with recruitment and data analysis. Community partners were offered compensation for their time at $15/hour. The University of Pittsburgh IRB approved this study as exempt, given it was anonymously conducted and any temporary identifiers were kept confidential and deleted permanently at the completion of interviews. In order to ensure anonymity of participants, community partners agreed with verbal informed consent rather than written consent, and our verbal consent script was approved by the University of Pittsburgh IRB.

We chose qualitative anonymous in-depth interviews in order to enhance insight into this poorly understood topic. Qualitative methods are ideal for understanding the experiences and beliefs of a specific population in their own voices and through their own narratives without predetermined investigator limitations or assumptions [22, 23]. Due to criminalization of exchange sex, we chose anonymous individual interviews and obtained a waiver of signed consent to reduce potential harm to participants from involvement in the study. While we kept telephone numbers or emails of participants in order to schedule and conduct interviews, after interviews were completed or three failed attempts to schedule an interview, contact information was deleted permanently. Any voicemails left on the study voicemail or sent to the study email were also deleted permanently, thus our participants remain anonymous. Our community partners reviewed our study materials including semi-structured interview guide, and recruitment flyers/social media messages and provided input and suggestions for revisions. (Additional files 1, 2, 3, 4, 5 and 6) Community partners agreed with the open-ended style of semi-structured individual interviews and recommended social-media based recruitment. Recruitment was thus multimodal, utilizing twitter profile, flyers, and purposive recruitment by community partners who referred personal contacts to the study. We also used snowball and respondent driven sampling, where participants could refer others to the study and receive $5 compensation for up to three referrals [24].

People were eligible for the study if they were English-speaking, 18 years or older, and had current or history of sex in exchange for money, favors, goods or services. Participants were not eligible if incarcerated or in jail at the time of the interview. We intentionally did not restrict participants based on self-identified gender or race in order to capture potential thematic similarities despite a sample population with heterogeneous gender and racial identities [23]. We used telephone calls or Zoom-based calls to conduct interviews from March 2020 to February 2021. A single trained interviewer conducted all interviews which started with a demographic questionnaire. (Additional file 7) The interviewer is experienced with clinical care of marginalized populations and with qualitative interviewing. During the demographic questionnaire which included age, self-described race and gender, we also asked an open-ended question, “How often in a month do you engage in sex for money, favors, or other goods/services?” where participants could indicate if they no longer exchanged sex. Participants self-described a term for the practice of exchange sex, due to the heterogeneity of terms among researchers and community members [25]. Interview topics included participant experiences and recommendations regarding health care services such as general healthcare, sexual health such as STD/HIV prevention, contraception, and pregnancy care including abortion services. After completing the interview, we provided educational materials about STDs, HIV risk, and PrEP. We provided resources to find a local PrEP provider, mental health resources, and legal resources.

Interviews were recorded and transcribed verbatim. Transcripts were edited to remove personal or geographical identifiers, and interview recordings were destroyed after transcription to preserve confidentiality. Through iterative feedback from community partners, we increased participant compensation from $15 per interview to $50 per interview. Before this feedback suggesting higher compensation, we had compensated two participants with $15. After the feedback, twenty participants received $50. Because no contact information is stored for participants, we could not contact prior participants to give them the additional compensation. All compensation was provided through gift cards. We planned to stop recruitment after achieving thematic saturation, which occurs when no new themes are identified during interviews and serves as an indicator that the sample size is robust to address the research question [22].

We used NVivo software to organize our analysis and used an open or “editing” approach to create a codebook [22]. While a general sense of thematic saturation was assessed by the single researcher conducting interviews, the interviewing researcher worked alongside three other researchers in order to create the codebook. Of the coding team, two of the researchers had read the majority of the interviews and participated in the transcription work. Thus, our coding team had a good understanding of the content of all the interviews when coding began. The four researchers independently coded 5 interviews, met to review independent codes, resolved discrepancies, and created a final codebook. A single researcher used this codebook to code all twenty-two interviews, and if any new codes were felt necessary we planned to review with the larger research team for agreement however no new codes were needed. We used inductive thematic analysis to identify themes, [23, 26]. No inter-rater reliability of codes was assessed, instead, coding updates and overall themes and subthemes were reviewed in detail with the full coding team which discussed the analysis and interpretation of the coding and agreed with the findings [23]. Additionally, findings were presented to members from our partner organizations who corroborated that the findings rang true to their experiences and perspectives [21]. Four community partners reviewed themes. We provided $50 for community partners who participated in thematic analysis review. These community partners confirmed that the themes rang true with their own experiences and expertise.

## Results

Twenty-two people participated in the study (Table 1). Most self-identified as non-white race with a median age of 26.5 (20–66 years). Participants had a variety of self-described genders, nine (40.9%) identified as female, nine (40.9%) identified as male and four (18.18%) identified as non-binary. While we did not ask for HIV serostatus in our demographic questionnaire, one participant disclosed HIV positive serostatus during their semi-structured interview. We achieved thematic saturation after 17 interviews and continued completing an additional five interviews out of a desire to respect participant’s interest in joining the study and avoid any trauma from perceived rejection or exclusion [22]. During our analysis of the data, we identified five themes which are discussed below.

- 1. Appreciation of HIV risk and prevention strategies grew from information accumulated over time.

**Table 1:** Demographic characteristics

Characteristic	Median (Range)
Age	26.5 (20–66)
Frequency of exchange sex in one month^a^	11.5 (2–60)
Race, self-described	N (%)
Gender, self-described	
Black	15 (68.18%)
Female/nonbinary – 1	1 (6.67%)
Female	8 (53.33%)
Male	6 (40%)
Multiracial	3 (13.63%)
Non-binary	2 (66.67%)
Human/feminine	1 (33.33%)
Transman	1 (33.33%)
Male	1 (33.33%)
White	3 (13.63%)
Non-binary/transmasculine	1 (33.33%)
Female	1 (33.33%)
Male	1 (33.33%)
Pacific Islander	1 (0.05%)
Male	1 (100%)
HIV positive, self-disclosed	1 (0.05%)

^a^*N* = 18, four participants did not endorse current exchange sex at the time of interview

HIV risk was commonly acknowledged among participants; as one participant articulated: “HIV is one of my biggest concerns and one of the biggest risks when you engage in this business. (Participant #7)” Participants often described that as they got older, gained more experiences, and learned more from others, their appreciation of HIV risk grew more serious over time. They described themselves as having been “impulsive (Participant #2)” and relatively naïve or ignorant of the risks when younger. They attributed some of this to a lack of education about STDs including HIV while in school. They discussed that with time they were able to learn more about STDs and HIV from non-school sources such as sexual health clinics. One participant described,

When I was younger I didn’t learn about how it’s transmitted. I didn’t learn about what happens if you do get it. I didn’t learn about prevention. It was at one of the clinics. I just popped in and they were like, oh, due to your history, you should consider taking PrEP. (Participant #3)

Many participants also explained that they felt more shame when they were younger related to exchange sex and thus would not acknowledge risk of HIV. They described how they learned from their experiences and the experiences of others. One participant explained that after having a friend acquire HIV, they became more aware of the risk and the risk itself felt severe, “I felt like I could get it, like it hits close to home… We slept with some of the same people. (Participant #9)” Another participant discussed that after being diagnosed with an STD they became aware of the risk of HIV acquisition (Participant #1).

Participants also shared that their growing awareness of HIV risk was not only related to their personal health but also the risk to their income. Participants feared that acquiring HIV would negatively impact their ability to perform their work. Indeed, many noted that acquiring any STD, including HIV, may compromise their ability to perform their job and receive financial compensation. This fear of losing work enhanced STD and HIV awareness and risk perception.

Participants described obtaining information about HIV in a fragmented process from varied sources. One participant discussed learning of testing, treatment, and barrier methods while attending a gay pride festival. Another described how, during prior imprisonment, they learned from other inmates to use latex gloves for barrier protection given the lack of condoms. Participants also described learning about HIV and prevention from clients, augmenting this information with their own research, “when I would have a client even if he was older than me, if he said things that didn’t sound right, I would go research it. (Participant #5)” Many participants described using the internet but fearing that the information may not be reliable. One participant explained that they preferred to find information that came from “a trusted facility, a place of higher learning like a medical facility. (Participant #10)” Many participants utilized community resources such as the library to find books on the subject. One individual discussed that a local business advocated for HIV awareness which helped them understand more about the disease. A few participants identified that celebrities and television resources were useful, and others used informational social media platforms. Others learned about HIV risk and mitigation strategies from in-person or on social media-based peer networks. Some participants served as a source of knowledge for their peers, one individual described that,

Most of the guys who do the same thing that I do are chancing on luck, maybe they are crossing their fingers that they won’t contract the disease. They don’t take the same precautions as much as I do. And that’s a big risk. I try to talk to these guys. (Participant #15)

- 2. PrEP information came from a variety of sources with mixed messages and uncertain credibility.

Participants had heterogeneous perceptions of and experiences with PrEP. One participant had never heard of PrEP (Participant #17). A few participants were taking PrEP at the time of the interview. Like their information regarding HIV risk, knowledge of PrEP also came from a variety of sources. Some discussed that information came from health care providers, either being counseled about PrEP after treatment for frequent STDs, or after disclosing sexual minority or exchange sex status. Several participants described learning about PrEP from flyers and brochures in emergency room or outpatient settings, saying that brochures were factually helpful because they could “…seek more information about it, and how do you take it, how many days do you take it. (Participant #14)” Other participants discussed learning of PrEP from peers, friends, or other colleagues. Other participants discussed learning about PrEP from bus signs, television commercials, or internet and social media avenues. Television commercials that included diverse racial, gender or sexual identities created a positive sense of inclusion and access to PrEP.

Participants described some mixed messages they received regarding PrEP. For example, many participants interpreted advertisements and media messaging to indicate that PrEP was “only for gay men, (Participant #22)” or people in same sex relationships. There was also confusion between PrEP and post-exposure prophylaxis (PEP) with several participants revealing during the course of the interview that they were describing PEP rather than PrEP and admitted that they may not have understood what PrEP was and how it differed from PEP. Others described hearing about various risks and side effects and voiced concerns about possible infertility, nausea, or uncertain long-term side effects given the newness of the medication. As one participant noted, “There are so many myths surrounding it. And because I don’t have the correct information, I don’t think I’m gonna use PrEP. (Participant #13)” Several also expressed mistrust in science and the medical system: One participant explained, “You know, science, I can’t trust what these people are telling me. (Participant #10)”.

- 3. Decision-making about use of PrEP was relative to other behavioral decisions regarding exchange sex.

Our study participants described how much of their decision-making regarding whether to take or continue PrEP related to choices they made regarding exchange sex. This is demonstrated in one participant’s explanation of PrEP after describing it as “life-saving,” going on to say, “when I deal with these clients who want to have sex without condoms—they might actually pay you less if you use condoms, or pay you more when you are not using them… it’s kind of tempting. I feel like the drug has really saved me in such scenarios because when I took the drug, I felt safe even though I wasn’t using condoms. (Participant #14)” Participants of all genders discussed risks with condom negotiation with clients such as physical violence or decreased compensation. Some participants valued PrEP because it gave them freedom not to use condoms, preferring to get frequently tested and take medication if diagnosed with other STDs. Others had used PrEP and discontinued the medication when they stopped practicing exchange sex, for example entering a monogamous relationship or transitioning to non-contact based sexual services (e.g. pornography or selling paraphernalia of sex such as undergarments).

Participants also discussed how their uncertainty, and associated lack of confidence, on how PrEP worked to prevent HIV influenced their willingness to use it. Given the cost of the medication and the work of taking a daily pill, participants considered the value of PrEP in light of their uncertainty of its benefit. To explain this, many participants contrasted their perception of PrEP with their views and use of condoms.

Many participants described that they preferred male or female condoms to PrEP, because condoms avoided some of the unpleasant features of PrEP (e.g. daily pill administration, cost concerns). They also believed the physical barrier of condoms to be more reliable or “sure.” This is demonstrated in this participant’s reasoning: “I don’t believe that it [PrEP] will protect me, so I don’t use them. It’s just a feeling, I find it difficult to trust drugs. I’d rather use one protection that I’m very sure—condoms. (Participant #11)” The uncertainty regarding how PrEP works is reflected in the following quote: “By nature of disease theory, you get infected or it’s transmitted… a drug will protect you from that? It’s not a sure thing. I don’t understand how that would work ensure I don’t get the disease. (Participant #11)” Others noted that while they understood the concept of PEP—taking medications to prevent HIV transmission in the context of an unprotected exposure—they did not perceive the benefit when risk was already mitigated with other protective measures such as condom use. As one participant explained the perceived difference between PEP and PrEP:

Because you take PEP after. Where PrEP is taken before. So when you take PEP you are sure there is something it is preventing. You only take PEP after you have messed up and you have to correct the situation. You can take PrEP and then you fail to mess up and you have loaded your body with unnecessary drugs. (Participant #16)

Other participants echoed this sentiment, indicating that they did not want to take medications “for no reason. (Participant #2)”.

- 4. The multi-step process of obtaining PrEP presented multiple potential barriers.

Participants described the multiple steps needed to obtain PrEP: getting an appointment with a health provider who prescribes PrEP, feeling comfortable at the appointment to discuss PrEP, having the funds or insurance coverage to fill the prescription, and facing various challenges at the pharmacy when picking up the prescription. Access to care was a concern and cost concerns were key barriers for both accessing health care and filling a prescription. Some participants described not being able to see a provider due to limited availability during the pandemic. Some described not using care due to lack of health insurance and thus not being able to initiate the process to obtain PrEP. One participant expressed appreciation that a clinic providing low-cost care to people without insurance allowed them to obtain PrEP (Participant #10). Another participant disclosed that cost and insurance coverage are also barriers to filling the prescription, “I know a lot of people tell me that their health insurance won't cover it, or that they cover it, but the copay is so high that it's cost prohibitive. (Participant #12)”.

In addition to the cost of PrEP, participants discussed the challenge of overcoming stigma, bias, and racism when interacting with both medical clinics and pharmacies. They described stigma faced when disclosing their experiences with transactional sex. For example, a participant described,

I had an encounter with a nurse because I had gone for an HIV test. I told her I’m a sex worker. Immediately she assumed that I’m HIV positive, and she didn’t ask me why I’m there, she just told me I’m going to have to get on the ART. I felt her assumption affected me, she just assumed rather than asking me. (Participant #14)

Other participants described the compounded impact of this stigma with racism. As the above participant shared, “When you are going to get tested for STDs or at the pharmacist when I buy condoms, at times they look at you in a certain manner. They can discriminate because I’m a black person, they don’t want to give you the service that you are seeking. (Participant #14)” This participant explained that they would not restart PrEP because they feared experiencing racism again, stating they are, “still worried about this… they are not going to give me PrEP because of my skin color. (Participant #14)” Another participant shared a similar experience of feeling discrimination at a pharmacy when picking up PrEP: “…people know my skin color… at times I will go there to pick up a tablet before meeting a client, and they will tell me they are out of stock. But probably some quiet guy comes in and gets the medicine. So I feel they could be discriminating against me. (Participant #19)”.

People frequently described that these experiences of racism and stigma prevented them from seeking care in the future or from disclosing their practice of exchange sex. One participant explained that.

When you get that from a healthcare provider it is kind of hurtful because you don’t know where you’re supposed to run to. You feel isolated and neglected because this is a place you have gone to feel safe when society has already judged you. You want to be able to share information about what you are doing, but when you visit any other hospital setting the bad experience keeps coming back to your mind. (Participant #21)

There were also multiple pharmacy-related barriers to PrEP use. Many participants discussed that they had interruptions in their PrEP use because the pharmacy was out of stock, and one participant noted they were not able to obtain PEP because a hospital pharmacy was out of stock. One participant noted that interruptions in PrEP use are, “heartbreaking (Participant #7)”. For this participant, PrEP provided a way to overcome HIV risk, and without PrEP clients were too risky. The participant declined clients when not using PrEP, thus suffering a loss of income when already struggling with financial insecurity. Some participants described that if they were travelling to a rural area, for example to the town of their family of origin, they would find the pharmacy in their hometown did not carry PrEP. They worried that the pharmacy didn’t carry PrEP due to the stigma associated with its use. The participant explained that they would have to, “stock-up (Participant #3)” when visiting home.

- 5. Healthcare providers were seen as powerful facilitators to PrEP utilization.

Several participants described their relationship with their providers as facilitators to PrEP utilization. One participant discussed that they had been able to obtain PrEP easily due to reliable providers, “Surprisingly, I’ve had no barriers… My doctor writes prescription every three months that I can refill every month. (Participant #12)” Other participants noted that a trusting relationship with their physician or nurse was a facilitator to PrEP utilization. One participant described feeling medical mistrust of the overall medical system, but that he was willing to learn about PrEP from a provider with whom he had developed a trusting relationship and over time. This participant explained the features that made this provider trustworthy,

I will say this. I’ve never had a doctor before this one that I can email in the middle of the night, if need be, if something happened. And know that I’m going to get response within 24 hours. Okay, that’s number one. It’s the type of personal interaction that you have with somebody that allows you to feel trustworthy. She really, in my opinion, hand-held me, walked me through, took the time out of her day. There were times when I didn’t even have an appointment however I would be able to have her personal email address, have her social media account. I can talk with her at any time, I have her phone number. That for me was the decision making in regards to, you know, being able to trust her. (Participant #10)

The participant explained that within this trusting relationship with their doctor, they were able to have repeated discussions about PrEP and ultimately chose to take PrEP while in a serodiscordant relationship.

Participants recommended that healthcare providers use their position to adjust the care continuum for PrEP, including enhancing awareness among people who have exchange sex and improving comprehensive sex education. Several participants voiced a preference to hear information about PrEP from their health care providers and noted that increased awareness was needed. As one participant stated, “…that is a gap that health providers have to fill in and give this information to sex workers in order to keep the number of HIV patients low. (Participant #7)” Another participant suggested that all clinic staff can provide PrEP information to increase awareness—“not only the doctors but also the nurses. (Participant #14)” Another participant suggested that holistic sex education should be a part of PrEP care in sexual health clinics. They described that this comprehensive sex education is critical to sexual health including PrEP use,

There should be a big push for us to be talking about relationships and sexual safety and sexual habits. A lot of people just get the PrEP without education. So that's my issue. You got to give them both. ...education based on healthy relationships...what you are willing to do, what you are not willing to do. How you can talk to your partner and address them safely... and in a way that gets your voice heard so you feel like you matter. (Participant #5)

## Discussion

People who have exchange sex constitute a highly stigmatized group that experiences barriers to potentially beneficial STD/HIV prevention services such as PrEP. Participants described that understanding their HIV risk was a complex, fragmented, cumulative process over time. They shared that obtaining knowledge about PrEP was also complex, informed by mixed-messages from varying sources and medical mistrust. When making decisions regarding use of PrEP, participants considered the impact of PrEP use on income from exchange sex and their understanding of disease prevention through medications or through condoms. Participants also perceived the process to obtain PrEP to be overly complex with barriers such as stigma, racism, prohibitive cost of medication, and poor access to healthcare. They recommended that healthcare providers streamline this process and increase access to PrEP and holistic sex education.

Our participants’ description of variable and cumulative understanding of HIV risk corroborates other studies examining HIV risk awareness among populations that face higher risk of HIV acquisition. A qualitative meta-synthesis of studies investigating HIV risk awareness and testing among at-risk groups including people who exchange sex, people who are incarcerated, or men who have sex with men, similarly identified lack of awareness regarding HIV testing, diagnosis or treatment as a barrier to HIV prevention [27]. Surveys among men who have sex with men in Toronto with objective criteria for high HIV risk showed correlation between low self-perceived HIV risk and reduced awareness of, interest in, or access to PrEP [28]. Our participants similarly described how perception of their HIV risks influenced decisions about PrEP. A study in Baltimore, MD identified that “female sex workers” (FSW) have limited awareness of prevention strategies and would be interested in PrEP [29]. Our participants’ interest and consideration of PrEP was nuanced and influenced by other risk and logistical considerations.

Our study illustrates decision-making regarding PrEP utilization accommodates personal preferences for broader risk/benefit considerations (e.g. higher income from sex without a condom) and includes concerns such as medical mistrust, and avoidance of medication side effects. A PrEP intervention trial among female sex workers (FSW) in Baltimore, MD described similar barriers such as medication side effects and medical mistrust [30]. While those researchers also described barriers such as difficulty obtaining venipuncture for recommended testing before initiation of PrEP, our study did not capture these barriers, which may be due to the heterogeneity of our participants while the Baltimore study included FSW with a high proportion of prior or current injection drug use [30]. Another study showing similar results to our study involved women who use drugs in a safe syringe program. Among women seeking care at a safe syringe program, while initial PrEP use was high at 66.3% (63 of 95 women), after 24 weeks only 42 women utilized PrEP (44.2%) [18]. Similar to our findings, this study’s participants had nuanced decision making around PrEP influenced by individual daily risk assessment regarding housing security and drug misuse with fluctuating levels of HIV risk perception [19]. Different from our study, participants of the safe syringe program revealed that they could sell their PrEP prescription obtained through the study to local predatory pharmacies to address their financial insecurity [19]. While our study participants discussed pharmacy barriers such as stigma, racism and lack of access, our participants did not discuss competing financial interests between keeping the PrEP for personal use or selling it to local pharmacies for income generation. In our study, participants discussed dislike for daily oral pills, thus the long-acting injectable formulation of PrEP using cabotegravir may be a promising avenue for HIV prevention among sex workers in the future, but at the time of our study it was not approved and no study participant mentioned non-oral formulations of PrEP [31].

Similar to prior qualitative work among people who have exchange sex, our study participants described troubling experiences of stigma and racism in healthcare settings that were barriers to PrEP use [32]. Different from our study, recent survey data among men who exchange sex identified that transportation difficulties and challenges discussing sex practices with a provider are also barriers to PrEP [33]. Our participants discussed that mistrust with healthcare provider was a barrier to PrEP but did not specifically identify transportation as an issue. Yet our findings provided a more comprehensive list of barriers, including access to healthcare, cost of medication with or without insurance coverage, issues with pharmacy, and lack of confidence in PrEP disease prevention. Our study may differ as it employed a community-partnered approach for recruitment, and it represents views of people who exchange sex with a variety of gender identities. Our study’s use of open-ended questions may account for participant’s expanded discussion of barriers to PrEP. One study among Canadian male sex workers who participated in a nurse-led PrEP program, described similar features of HIV risk awareness.

Participants that we interviewed for this study discussed experiences of violence and assault by clients and police officers. This is critical to understanding barriers to STD/HIV prevention among people who exchange sex, given that modeling has shown that elimination of police violence coupled with support to address long term effects of violence could prevent 24% (95% CI 8–45%) of HIV infections among female sex workers (FSW) over a decade [34]. Work among Black men who have sex with men in the Los Angeles area using a syndemic model shows that there is a synergistic effect of experiences of violence and increased risk of exchange sex practice, and additional work is needed to understand the intersection of violence, exchange sex and increased HIV risk [35]. Our study objective was to understand healthcare experiences among people who exchange sex, and while violence and intersection with police and carceral systems are also a crucial area to investigate, that was not the focus of our work. Through our community partnered approach, we discussed our findings and reviewed themes with community partners, and our team felt the area of violence and policing was not adequately investigated in our data and so we are not disseminating those comments from participants.

Limitations of our work include age range represented, lack of sexual identity reporting, and low compensation. Mandatory reporting laws in Pennsylvania require reporting of minors involved in exchange sex to state child services, and revealing a participant’s identity would not be ethical. Thus, we did not interview participants less than 18 years old. We recognize that this misses the perspectives and experiences of this key population as one in fourteen high school aged adolescents in Washington DC are estimated to participate in exchange sex [36]. Additionally, although an argument can be made that asking about sexual identity is valuable and may add insight to the data [37], our community partners, including LGBTQIA sexual health groups, did not recommend asking about sexual identity either in the interview or the demographic questionnaire. While some participants did discuss their sexual identity at times, not all participants reported this in their interviews, so we are not able to comprehensively report about sexual identity and its intersection with healthcare and exchange sex. Finally, as mentioned in the methods, our initial compensation was too low at $15 per interview, and although we did increase compensation to $50 it is likely that this compensation may also have been too low to attract a broader sample of individuals who engage in exchange sex. While studies suggest that community-advisory boards should determine reasonable compensation for studies among marginalized populations, and our study did employ community-partnered methods, COVID19 impacts may have altered what is deemed reasonable during our study and future work may benefit from increased compensation amounts [38]. Research activities were also halted for two months at the start of the COVID19 pandemic, and social distancing requirements prevented any in-person recruitment. This may have enhanced social media and flyer-based recruitment, and may have overall limited our ability to recruit from a wide variety of potential participants. Strengths of this work include its heterogeneous population and community-partnered approach. Despite the limitations, our work was able to identify common themes among a very diverse group of participants that include a wide age range, and a variety of gender and race identities. Commonality despite these differing identities may strengthen our findings as potential avenues for future prevention efforts.

## Conclusions

Our findings have important clinical, policy, research and educational implications. Our work provides a variety of strategies that are potential avenues to effect change. Participants recommended that healthcare providers should create personal relationships with patients to establish trust, and should promote awareness of PrEP. Counseling approaches may also wish to acknowledge and address the balancing of various risk/benefit considerations that include exploring individual’s prioritization of concerns and brainstorming supports and mitigation strategies for other risks including interpersonal violence and stigma/bias. As suggested by participants, messaging regarding HIV risk prevention strategies including PrEP should use multiple communication platforms and modes such as social media outlets and use of peer networks. Additionally, messaging should be inclusive of a variety of gender identities and sexual orientations. And, as participants of our study described receiving healthcare in the emergency room for PEP, embedding preventive and sexual education services integrated into emergency department services and resources, including a stronger PEP to PrEP pathway, may facilitate entry and engagement in these preventative services.

## Supplementary Information

**Additional file 1. **

**Additional file 2. **

**Additional file 3. **

**Additional file 4. **

**Additional file 5. **

**Additional file 6. **

**Additional file 7. **

## Data Availability

Original data can be requested from the corresponding author but may be subject to IRB review and data use agreement.

## Abbreviations

STDs: Sexually transmitted diseases
HIV: Human immunodeficiency virus
PrEP: Pre-exposure prophylaxis
PEP: Post-exposure prophylaxis
