# Supplementary material for: HIV awareness, pre-exposure prophylaxis perceptions and experiences among people who exchange sex: qualitative and community based participatory study
Source: BMC Public Health. 2022 Oct 1;22:1844. doi: 10.1186/s12889-022-14235-0 (PMC9526910; doi:10.1186/s12889-022-14235-0)
Supplement: Supplementary file 1 — Additional file 1. [file 12889_2022_14235_MOESM1_ESM.pdf]

Interview Guide:

Core Questions for Individual Interviews

1. **What interested you in joining this study?**
2. **What term do you use to describe when you are exchanging sex for money, favors, goods or services?**
3. In Person vs Telephone:
   a. Telephone: **Tell me about the role sex has had in your life?**
   b. In-Person: Interviewer uses a sheet of paper with a single line drawn in the middle. **Imagine the beginning of the line is your birth, and the end of the line is the present. Can you write in the first time you had sex? How about the first time you _(use participant's terms for exchanging sex)?**

4. **What kinds of worries do you have when you are ____**(use participant's terms for exchanging sex)**?** What do you do to protect yourself from these worries? If STD not mentioned: What do you think about sexually transmitted diseases? If HIV not mentioned: What do you think about HIV? What about pregnancy?

5. **Tell me how you get your health care?** What barriers do you experience to getting resources that you need? What kind of care have you gotten to protect you from or treat you for sexually transmitted disease? How about any care for birth control or pregnancy?
6. **Tell me about some of the interactions or communication you have had with health care providers about sex-related issues?**
7. **Have you heard about pills to prevent HIV, it's also called "PrEP"?** What have you heard?
8. **What advice do you have for improving how health care providers give care to people who ___** (use participant's terms for exchanging sex)**?**
9. **How has the COVID19 pandemic affected your ___** (use participant's terms for exchanging sex)? What do you do to protect yourself from COVID19? If healthcare not mentioned: How does the pandemic change your relationship with healthcare?
10. **What other information do you think is helpful for me to know regarding this topic for this study?**
11. **If there is anyone else that you think might want to share their story to our study team, please have them contact us at yourstory@upmc.edu or 412-641-3712**
