# Supplementary material for: HIV awareness, pre-exposure prophylaxis perceptions and experiences among people who exchange sex: qualitative and community based participatory study
Source: BMC Public Health. 2022 Oct 1;22:1844. doi: 10.1186/s12889-022-14235-0 (PMC9526910; doi:10.1186/s12889-022-14235-0)
Supplement: Supplementary file 2 — Additional file 2. [file 12889_2022_14235_MOESM2_ESM.pdf]

Have you ever:

- Traded sex for rent?
- Traded sex for food, transportation, or other services?
- Had sex in exchange for money?
- Traded sex for drugs?

If you answer yes to any of these questions, your feedback is requested!

A University of Pittsburgh research study is exploring the relationship of healthcare to sexual behaviors like these, and your opinions matter!

Participants are anonymously interviewed. A $50 gift certificate will be provided as appreciation for your time

Email YourStory@upmc.edu or Call 412-641-3712 to join the research team!

yourstory@upmc.edu
412-641-3712

yourstory@upmc.edu
412-641-3712

yourstory@upmc.edu
412-641-3712

yourstory@upmc.edu
412-641-3712

yourstory@upmc.edu
412-641-3712

yourstory@upmc.edu
412-641-3712

yourstory@upmc.edu
412-641-3712

yourstory@upmc.edu
412-641-3712

yourstory@upmc.edu
412-641-3712

yourstory@upmc.edu
412-641-3712
