# Supplementary material for: HIV awareness, pre-exposure prophylaxis perceptions and experiences among people who exchange sex: qualitative and community based participatory study
Source: BMC Public Health. 2022 Oct 1;22:1844. doi: 10.1186/s12889-022-14235-0 (PMC9526910; doi:10.1186/s12889-022-14235-0)
Supplement: Supplementary file 3 — Additional file 3. [file 12889_2022_14235_MOESM3_ESM.pdf]

Have you ever:

- Traded sex for rent?
- Traded sex for drugs?
- Traded sex for food, transportation, or other services?
- Been forced to have sex for money?
- Chosen to have sex for money, goods or services - because it is your profession?

If you answer yes to any of these questions, your feedback is requested!

A University of Pittsburgh research study is exploring the relationship of healthcare and these behaviors. Your opinions matter! Participants are anonymously interviewed and receive a $50 appreciation gift certificate.

CLICK HERE to sign up!

You can also email **YourStory@upmc.edu** or call **412-641- 3712** to join
