# Supplementary figures and images for: HIV awareness, pre-exposure prophylaxis perceptions and experiences among people who exchange sex: qualitative and community based participatory study
Source: BMC Public Health. 2022 Oct 1;22:1844. doi: 10.1186/s12889-022-14235-0 (PMC9526910; doi:10.1186/s12889-022-14235-0)

# **Your Story**

A University of Pittsburgh Research Study

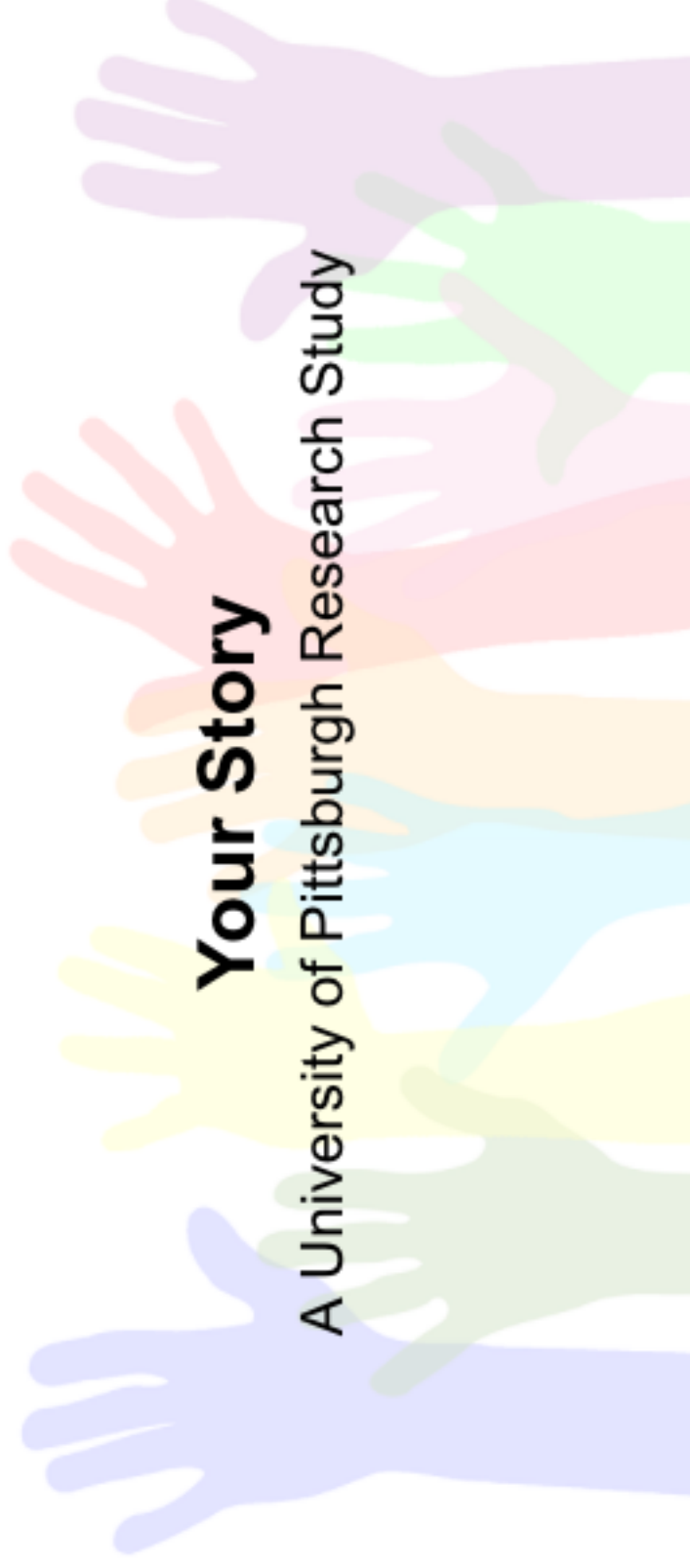

Supplement: Supplementary file 4 — Additional file 4. [file 12889_2022_14235_MOESM4_ESM.pdf]
