# Supplementary material for: HIV awareness, pre-exposure prophylaxis perceptions and experiences among people who exchange sex: qualitative and community based participatory study
Source: BMC Public Health. 2022 Oct 1;22:1844. doi: 10.1186/s12889-022-14235-0 (PMC9526910; doi:10.1186/s12889-022-14235-0)
Supplement: Supplementary file 5 — Additional file 5. [file 12889_2022_14235_MOESM5_ESM.pdf]

Age?

Are you 18 years or older?

- ◯ Yes
- ◯ No

History of exchanging sex?

Do you have a history or current engagement with sex in exchange for money, favors, goods or services?

- ◯ Yes
- ◯ No

Incarceration?

Are you currently in jail or prison?

- ◯ Yes
- ◯ No

Language?

The interviewers only speak English, are you comfortable speaking in English?

- ○ Yes
- ○ No

Compensation

Thank you for being interested in sharing your thoughts and experiences. We are compensating participants with Amazon e-gift cards. The cards are sent to a cell phone number or an email. Just making sure that is OK with you!

- ○ I am comfortable with receiving an Amazon e-gift card to my email or phone.
- ○ I am not comfortable with receiving an Amazon e-gift card. Here's why: __________

You can receive $5 for the participation of anyone who you refer to our study. Please choose a unique identifier so we know who should receive the $5 referral compensation. Share this unique identifier with anyone you refer to the study.

- ○ What is your unique, anonymous identifier? It shouldn't be related to your name or any other identifying feature. It can be an alpha-numeric, e.g. "123AB" __________

Contact information?

To keep your identity secret, your contact information will be deleted after you are emailed or telephoned.
Please enter your preferred contact information:

- ○ __________ Email

○ ________ Phone

Powered by Qualtrics
