# Supplementary material for: HIV awareness, pre-exposure prophylaxis perceptions and experiences among people who exchange sex: qualitative and community based participatory study
Source: BMC Public Health. 2022 Oct 1;22:1844. doi: 10.1186/s12889-022-14235-0 (PMC9526910; doi:10.1186/s12889-022-14235-0)
Supplement: Supplementary file 6 — Additional file 6. [file 12889_2022_14235_MOESM6_ESM.pdf]

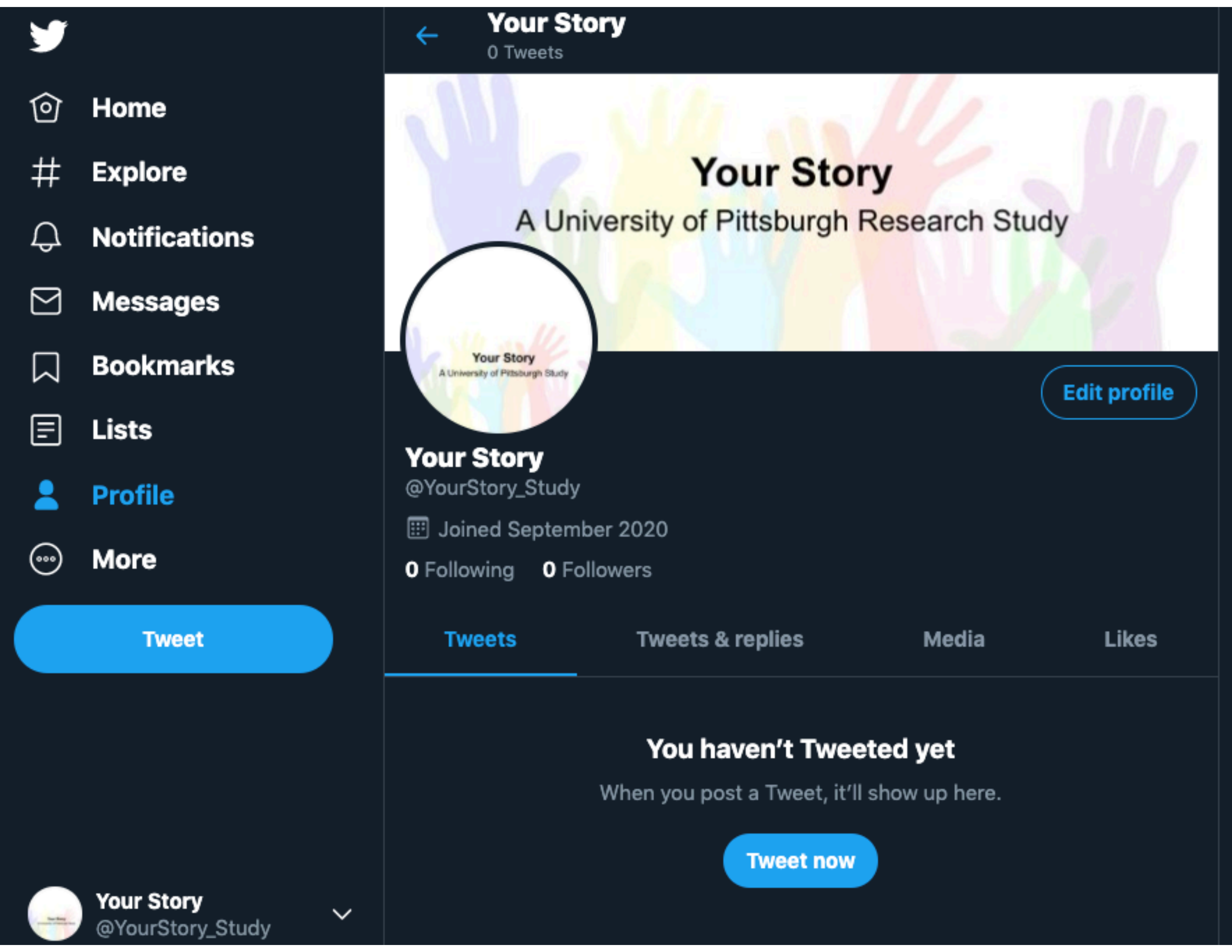
Your Story
0 Tweets
Home
Explore
Notifications
Messages
Bookmarks
Lists
Profile
More
Tweet
Your Story
A University of Pittsburgh Research Study
Edit profile
Your Story
@YourStory_Study
Joined September 2020
0 Following
0 Followers
Tweets
Tweets & replies
Media
Likes
You haven't Tweeted yet
When you post a Tweet, it'll show up here.
Tweet now
Your Story
@YourStory_Study
