# Supplementary material for: HIV awareness, pre-exposure prophylaxis perceptions and experiences among people who exchange sex: qualitative and community based participatory study
Source: BMC Public Health. 2022 Oct 1;22:1844. doi: 10.1186/s12889-022-14235-0 (PMC9526910; doi:10.1186/s12889-022-14235-0)
Supplement: Supplementary file 7 — Additional file 7. [file 12889_2022_14235_MOESM7_ESM.pdf]

Demographic Section:

1. What is your age? __________

2. What is your gender?

- Male
- Female
- Transgender female
- Transgender male
- Genderqueer
- Self-described __________________________
- Prefer not to respond

3. What pronouns do you prefer?

- She/Her
- He/His
- They/Them
- All
- Prefer not to respond

4. How often in a month do you engage in sex for money, favors, or other goods/services?

____

5. What race do you identify with (select all that apply)?

- White
- Black or African American
- LatinX
- Native American
- Asian
- Asian Indian
- Pacific Islander
- Self-described _________________
- Prefer not to respond
